# Follistatin-Like 1 Protects against Doxorubicin-Induced Cardiomyopathy through Upregulation of Nrf2

**DOI:** 10.1155/2020/3598715

**Published:** 2020-08-01

**Authors:** Yintao Zhao, Jingjing Sun, Wei Zhang, Meng Peng, Jun Chen, Lu Zheng, Xiangqin Zhang, Haibo Yang, Yuan Liu

**Affiliations:** ^1^Department of Cardiology, The First Affiliated Hospital of Zhengzhou University, Zhengzhou, China; ^2^Department of Anesthesiology, The First Affiliated Hospital of Zhengzhou University, Zhengzhou, China

## Abstract

Doxorubicin- (DOX-) induced cardiomyocyte loss results in irreversible heart failure, which limits the clinical applications of DOX. Currently, there are no drugs that can effectively treat DOX-related cardiotoxicity. Follistatin-like 1 (FSTL1) has been reported to be a transforming growth factor-beta-inducible gene, and FSTL1 supplementation attenuated ischemic injury and cardiac apoptotic loss in mice. However, the effect of FSTL1 on DOX-induced cardiomyopathy has not been elucidated. We aimed to explore whether FSTL1 could prevent DOX-related cardiotoxicity in mice. Mice were intraperitoneally injected with a single dose of DOX to induce acute cardiotoxicity. We used an adeno-associated virus system to overexpress FSTL1 in the heart. DOX administration decreased FSTL1 mRNA and protein expression in the heart and in cells. FSTL1 prevented DOX-related cardiac injury and inhibited cardiac oxidative stress and apoptosis, thereby improving cardiac function in mice. FSTL1 also improved cardiomyocyte contractile functions in vitro. FSTL1 upregulated expression of nuclear factor (erythroid-derived 2)-like 2 (Nrf2) in DOX-treated hearts. FSTL1 was not capable of protecting against these toxic effects in Nrf2-deficient mice. In conclusion, FSTL1 protected against DOX-induced cardiotoxicity via upregulation of Nrf2 expression.

## 1. Introduction

Doxorubicin (DOX), a well-known and highly effective chemotherapy drug, can contribute to the development of cardiotoxicity and irreversible heart failure, which largely limits its clinical use [1]. Heart transplantation is the only approach to treating serious cardiomyopathy in patients that were administered with DOX [2]. The precise pathogenesis of DOX-related cardiotoxicity has not been completely elucidated, but several lines of evidence have suggested that oxidative damage and myocardial apoptotic loss are closely involved [3, 4]. Thus, finding a drug that can restrict DOX-induced cardiac apoptosis might be important in treating DOX-related cardiac injury.

Follistatin-like 1 (FSTL1) is an extracellular glycoprotein that plays key roles in cardiometabolic diseases. It has been reported that FSTL1 supplementation limits ischemic injury and cardiac apoptotic loss in mice [5]. Similarly, FSTL1 exerted therapeutic effects by decreasing ventricle mass [6]. Moreover, elevated plasma FSTL1 concentrations are closely correlated with brain natriuretic peptide release [7]. In addition, FSTL1 has been found to upregulate protein kinase B (PKB/AKT) activity in mice [5]. However, there have been no reports describing the role of FSTL1 in DOX-related acute cardiotoxicity.

Herein, we sought to examine the hypothesis that FSTL1 could prevent DOX-related cardiotoxicity in mice. The results showed that FSTL1 expression was decreased in DOX-treated hearts and cells. FSTL1 restoration attenuated DOX-related cardiac injury and dysfunction and suppressed cardiac apoptosis in mice. Moreover, we found that the protective effect of FSTL1 was dependent on the restoration of nuclear factor (erythroid-derived 2)-like 2 (Nrf2).

## 2. Methods

### 2.1. Reagents

Recombinant human FSTL1 (≥97% purity, as determined by HPLC) was obtained from PeproTech Co. (New Jersey, USA). Primary antibodies against FSTL1 (#ab11805, 1 : 1000), GAPDH (#ab181602, 1 : 1000), Nrf2 (#ab62352, 1 : 1000), and cleaved caspase-3 (#ab2302, 1 : 1000) were obtained from Abcam (Cambridge, UK). A mouse N-terminal brain natriuretic peptide precursor (NT-proBNP) assay kit was obtained from CUSABIO (#CSB-E05153m, Wuhan, China). DOX (#D1515) was provided by Sigma-Aldrich (MO, USA). A mouse cardiac troponin I (cTnI) ELISA kit was provided by CUSABIO (#CSB-E08421m). A mouse creatine kinase (CK) ELISA kit (#CSB-E14404m) and lactate dehydrogenase (LDH) ELISA kit (#CSB-E17733m) were also provided by CUSABIO.

### 2.2. Animal Studies

This study was approved by the Committee on Animal Care of the First Affiliated Hospital of Zhengzhou University and was carried out in accordance with the National Institutes of Health Guidelines on the Use of Laboratory Animals. All C57/BL6J mice (age: 12 weeks, 25-28 g, male) were provided by the Chinese Academy of Medical Sciences (Beijing, China). All mice (*n* = 48) were randomly divided into 4 groups (*n* = 12 in each group): normal saline (NS)+control (con), NS+FSTL1, DOX+con, and DOX+FSTL1. We used an adeno-associated virus (AAV9) system to overexpress FSTL1 in the heart. AAV9-FSTL1 and AAV9-*β*-gal were provided by Vigene Bioscience, and AAV9-*β*-gal was used as the control. To investigate the protective effects of FSTL1, mice in the FSTL1 group received a single tail vein injection of AAV9-FSTL1, and the control group received a single tail vein injection of AAV9-*β*-gal at a dose of 5 × 10^11^ particles 4 weeks before DOX injection [8–10]. Four weeks after AAV9 infection, all the animals were subjected to a single intraperitoneal injection of DOX (20 mg/kg) [11]. The efficacy of AAV9-FSTL1 was confirmed by Western blotting at the endpoint of the study. Two days after DOX treatment, all mice were sacrificed immediately via an intraperitoneal injection of sodium pentobarbital (150 mg/kg). We collected heart samples for biochemical analysis. To confirm the hypothesis that FSTL1 exerted cardioprotective effects by activating Nrf2, mice were intramyocardially injected with adenoviral genome particles (1 × 10^9^ plaque forming units) carrying small interfering RNAs targeting Nrf2 (siNrf2) in the left ventricle [3]. The efficacy of siNrf2 was confirmed by Western blotting. Seven days after intramyocardial injection, all mice were administered a single injection of DOX (20 mg/kg, once) as previously described. siNrf2 was obtained from Santa Cruz.

### 2.3. Hemodynamics Analysis

Left ventricle function was assessed by invasive hemodynamic monitoring. We exposed the left ventricle at the seventh intercostal space, and a 1.0 F catheter (SPR 839; Millar Instruments Inc.) was inserted into the left ventricle along the longitudinal axis. The data were analyzed using Labchart software. This experiment was performed in a blinded manner.

### 2.4. RNA Analysis

Total RNA samples were obtained from pulverized left ventricles with TRIzol reagent (Invitrogen). cDNA synthesis was achieved using the Superscript III reverse transcriptase kit (Invitrogen). Real-time PCR was performed using a LightCycler 480 SYBR Green master mix kit (Roche Diagnostics). GAPDH was selected as the internal control.

### 2.5. Western Blotting

Frozen heart samples were homogenized with RIPA lysis buffer. Proteins were loaded into 10% SDS-PAGE gels and then electrotransferred to Immobilon-FL transfer membranes (Millipore, IPFL00010). Then, the Immobilon-FL transfer membranes were incubated with primary antibodies against FSTL1 (#ab11805, 1 : 1000), GAPDH (#ab181602, 1 : 1000), Nrf2 (#ab62352, 1 : 1000) and cleaved caspase-3 (1 : 1000, #ab2302) [12]. Band intensity was scanned and analyzed by NIH ImageJ software, and GAPDH was selected as the internal control.

### 2.6. Antioxidant Assay and Determination of Cardiac Injury Markers

To detect cardiac oxidative damage, fresh heart samples were homogenized. Superoxide dismutase (SOD) activity was detected using a commercially available kit (Nanjing Jiancheng Bioengineering Institute, Nanjing, China). Lipid peroxidation products, including malondialdehyde (MDA) and 4-hydroxynonenal (4-HNE), were also measured as a reflection of DOX-related oxidative damage. The MDA assay kit was provided by Nanjing Jiancheng Bioengineering Institute, and the 4-HNE detection kit was provided by Abcam (#ab238538).

To detect cardiac injury markers, blood was collected and measured for cTnI, NT-pro BNP, CK, and LDH levels using commercial kits.

### 2.7. Apoptotic Cell Death Assay

Fresh heart samples were collected and assayed for myocardial apoptotic cell death using terminal deoxynucleotidyl transferase-meditated dUTP nick-end labeling (TUNEL) staining with an in situ apoptosis detection kit (Roche Diagnostics Ltd., USA). Fresh heart samples were homogenized, and the activity of caspase3 in the heart was examined using a caspase3 activity assay kit from Nanjing Jiancheng Bioengineering Institute.

### 2.8. Adult Mouse Cardiomyocyte Isolation and Mechanics Detection

Adult ventricular myocyte isolation was achieved by a temperature-controlled Langendorff system. The fresh heart samples were incubated with liberase (0.1 mg/ml, Sigma-Aldrich) for 25 min. Only rod-shaped cardiomyocytes with clear edges were selected for further detection in our study. The IonOptix™ soft-edge system (IonOptix, MA, USA) was used to assess cardiomyocyte contractile properties. Cardiomyocyte mechanical properties were assessed in more than 200 cells per group (*n* = 5, more than 40 cells per mouse).

### 2.9. Neonatal Rat Cardiomyocyte Culture

Primary neonatal rat cardiomyocytes (NRCMs) were prepared as previously described [13]. These cells were cultured in DMEM (Gibco, NY, USA) containing 10% fetal bovine serum (Gibco). Human recombinant FSTL1 was dissolved in PBS. NRCMs were subjected to DOX treatment (5 *μ*mol/l) for 24 h. To explore the effect of FSTL1, NRCMs were incubated with FSTL1 (5 *μ*g/ml) or the same volume of PBS. For RNA interference analysis of Nrf2, siNrf2 (Invitrogen) was used, and scrambled siRNA was used as a nonspecific control. NRCMs were treated with siNrf2 (50 nmol/l, 6 h) to achieve Nrf2 knockdown. To detect ROS production in NRCMs, cells were seeded in 96-well culture plates and then treated with DOX (5 *μ*mol/l) for 24 h. Then, the NRCMs were incubated with DCFH-DA (10 *μ*mol/l) at 37°C for 30 min, and ROS levels were examined by a microplate reader. Cell viability was assessed by a CCK-8 kit according to the manufacturer's instructions. Cells were also homogenized with PBS to measure the release of LDH after FSTL1 treatment.

### 2.10. Detection of Nuclear Nrf2 Activity

Frozen heart samples were homogenized with RIPA lysis buffer, and nuclear proteins in the heart samples were prepared using NE-PER™ nuclear and cytoplasmic extraction reagents. Then, Nrf2-binding activity was measured using the Nrf2 DNA-binding ELISA kit (TransAM Nrf2, Active Motif).

### 2.11. Mitochondrial Isolation and Complex Activity Detection

Fresh heart samples were homogenized, and mitochondria were isolated using a mitochondria isolation kit (#89801, Thermo Fisher Scientific). Myocardial mitochondrial complex activities were measured using the MitoCheck Complex I activity assay kit (#700930, Cayman), MitoCheck Complex II/III activity assay kit (#700950, Cayman), and MitoCheck Complex IV activity assay kit (#700990, Cayman).

### 2.12. Statistical Analysis

All data are shown as the means ± SD. Comparisons between multiple groups were performed using one-way ANOVA followed by a post hoc Tukey's multiple comparisons test. Comparisons among two groups were performed using two-tailed Student's *t* tests. Statistical significance was accepted at a value of *P* < 0.05.

## 3. Results

### 3.1. FSTL1 Was Decreased in DOX-Treated Hearts and Cells

**Figure 1:**
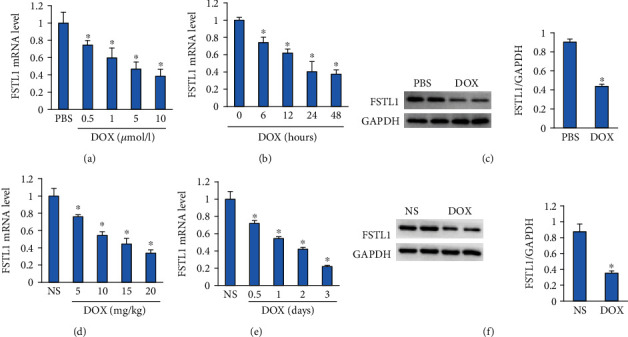
FSTL1 expression after DOX treatment. (a, b) The mRNA level of FSTL1 in DOX-treated NRCMs (*n* = 6). (c) Protein expression of FSTL1 in DOX-treated NRCMs (*n* = 6). (d, e) The mRNA level of FSTL1 in DOX-treated hearts (*n* = 6). (c) Protein expression of FSTL1 in DOX-treated hearts (*n* = 6). ^∗^*P* < 0.05 vs. the NS/PBS groups. The data were analyzed using two-tailed Student's *t*-tests.

We first measured FSTL1 mRNA in NRCMs treated with DOX for 24 h. Interestingly, DOX decreased FSTL1 mRNA levels in a dose-dependent manner (Figure 1(a)). DOX (5 *μ*mol/l) also decreased FSTL1 mRNA levels in a time-dependent manner (Figure 1(b)). FSTL1 protein expression in DOX-treated cells was reduced to approximately 48.7% of that in the PBS control (Figure 1(c)). Next, we detected FSTL1 mRNA levels in hearts that were treated with DOX for 2 days and found that FSTL1 mRNA levels in mice were also dose-dependently decreased after DOX treatment (Figure 1(d)). DOX (15 mg/kg) also decreased FSTL1 mRNA levels in a time-dependent manner in mice (Figure 1(e)). DOX injection also led to significant downregulation in FSTL1 protein expression in the heart (Figure 1(f)).

### 3.2. FSTL1 Overexpression Protected against Cardiac Injury in DOX-Treated Mice

**Figure 2:**
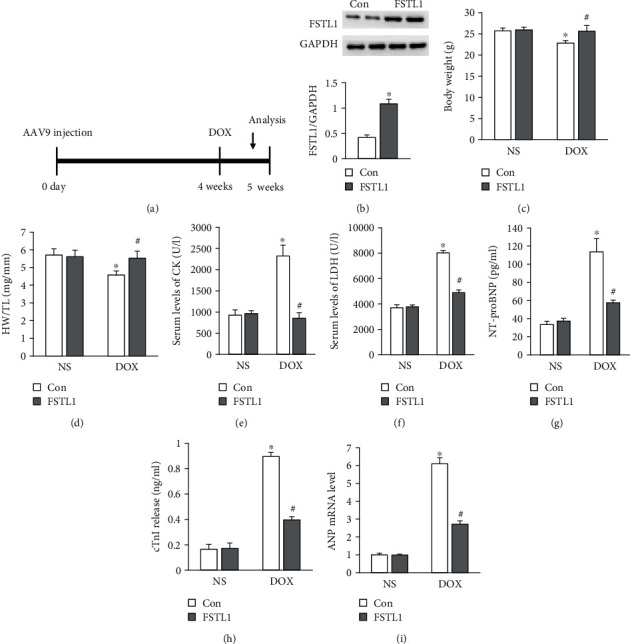
FSTL1 overexpression reduced myocardial injury in mice. (a) A diagram illustrating the study protocol. (b) FSTL1 protein expression in the heart (*n* = 6). (c, d) Body weight and heart weight/tibia length ratio (*n* = 12). (e, f) The levels of CK and LDH (*n* = 6). (g, h) The levels of NT-proBNP and cTnI (*n* = 6). (i) The mRNA level of ANP in the heart (*n* = 6). The data are expressed as the mean ± SD. ^∗^*P* < 0.05 vs. the NS/con group; ^#^*P* < 0.05 vs. the DOX/con group. The data were analyzed using one-way ANOVA, followed by Tukey's post hoc analysis.

AAV9 infection induced a 2.5-fold increase in FSTL1 expression in the heart (Figures 2(a) and 2(b)). DOX injection induced a decrease in body weight and the heart weight/tibia length ratio (Figures 2(c) and 2(d)). However, these pathological changes were abolished after FSTL1 overexpression in mice (Figures 2(c) and 2(d)). FSTL1 overexpression decreased the levels of plasma CK, LDH, NT-proBNP, and cTnI in DOX-treated hearts (Figures 2(e)–2(h)). FSTL1 overexpression also significantly decreased the mRNA level of atrial natriuretic peptide in DOX-treated mice (Figure 2(i)).

### 3.3. FSTL1 Improved Cardiac Function and Adult Cardiomyocyte Contractile Function

**Figure 3:**
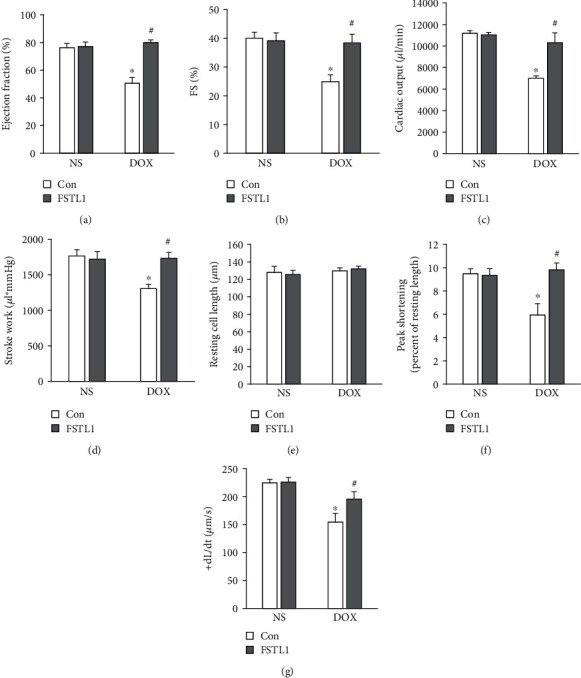
Effects of FSTL1 overexpression on DOX-induced cardiac dysfunction. (a, b) Ejection fraction and fraction shortening (*n* = 8). (c, d) Stroke work and cardiac output in mice (*n* = 8). (e) Resting cell length in isolated adult cells (*n* = 5). (f, g) Alterations in peak shortening and +dL/dt (*n* = 5). The data are expressed as the mean ± SD. ^∗^*P* < 0.05 vs. the NS/con group; ^#^*P* < 0.05 vs. the DOX/con group. The data were analyzed using one-way ANOVA, followed by Tukey's post hoc analysis.

Hemodynamic parameters were evaluated, and ejection fraction (EF), fraction shortening (FS), cardiac output, and stroke work were significantly decreased in DOX-injected mice compared with NS-injected mice in all groups. These markers were significantly preserved after FSTL1 overexpression in DOX-treated mice (Figures 3(a)–3(d)). Next, we detected the effect of FSTL1 overexpression on the contractile function of isolated single adult cardiomyocytes. FSTL1 overexpression did not affect resting cell length in any of the groups (Figure 3(e)). Adult cardiomyocytes isolated from DOX-treated hearts showed decreased contractile function, as indicated by alterations in peak shortening and the maximal velocity of shortening/relengthening (+dL/dt). However, FSTL1 supplementation restored cardiomyocyte contractile function in DOX-treated hearts (Figures 3(f) and 3(g)).

### 3.4. FSTL1 Attenuated DOX-Induced Oxidative Damage in Mice

**Figure 4:**
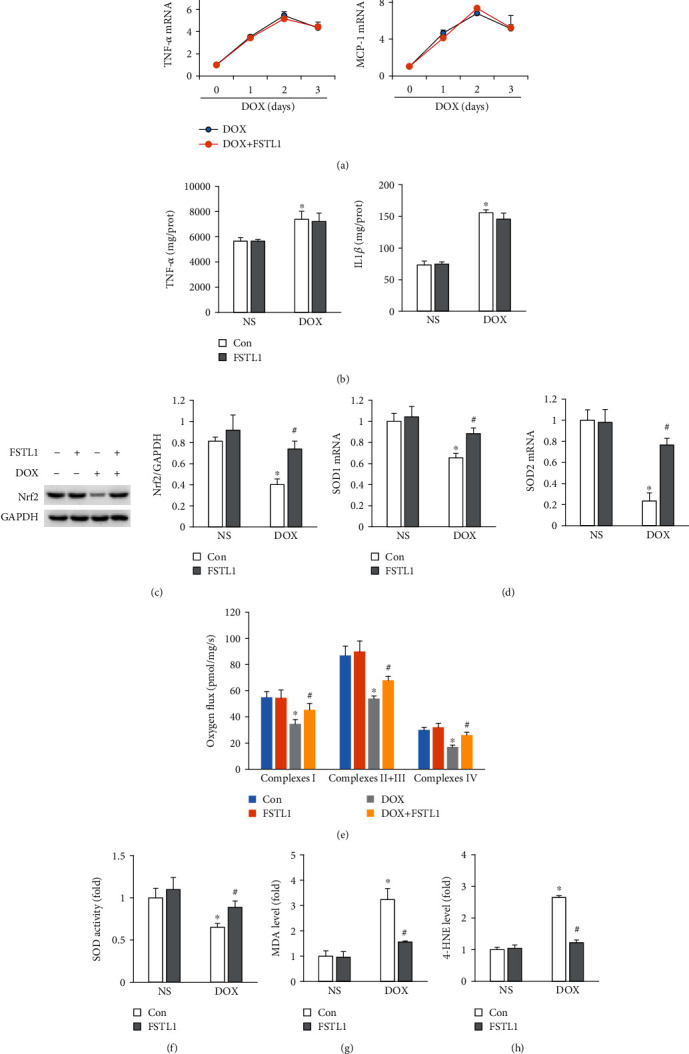
FSTL1 overexpression reduced DOX-induced myocardial oxidative damage. (a) The mRNA levels of TNF-*α* and IL-1*β* (*n* = 5 for each time point). (b) Myocardial TNF-*α* and IL-1*β* levels were determined by ELISA (*n* = 6). (c) Protein expression of Nrf2 in the heart (*n* = 6). (d) The mRNA levels of SOD1 and SOD2 (*n* = 6). (e) Mitochondrial complex activities (*n* = 6). (f) Alterations in SOD activity (*n* = 6). (g, h) MDA and 4-HNE levels (*n* = 6). The data are expressed as the mean ± SD. ^∗^*P* < 0.05 vs. the NS/con group; ^#^*P* < 0.05 vs. the DOX/con group. The data were analyzed using one-way ANOVA, followed by Tukey's post hoc analysis.

Proinflammatory mediators contribute to the development of acute DOX-induced toxicity [14]. We first measured tumor necrosis factor- (TNF-) *α* and monocyte chemotactic protein 1 (MCP-1) mRNA levels and found that FSTL1 did not decrease expression of these two proinflammatory factors (Figure 4(a)). We used ELISA to measure myocardial TNF-*α* and interleukin-1*β* (IL-1*β*) production. There was no difference in the concentration of TNF-*α* or IL-1*β* between the DOX and DOX+FSTL1 groups (Figure 4(b)). DOX decreased the protein expression of Nrf2, and FSTL1 overexpression largely restored the protein level of Nrf2 (Figure 4(c)). FSTL1 also increased SOD1 and SOD2 mRNA levels in DOX-treated hearts (Figure 4(d)). Next, fresh heart samples were collected to assess the electron transport chain. We showed that DOX treatment significantly impaired complex I, II+III, and IV activities in mice. However, FSTL1 supplementation restored these functions in mice (Figure 4(e)). We also measured total SOD activity and found that FSTL1 increased SOD activity in DOX-treated hearts (Figure 4(f)). DOX increased MDA and 4-HNE levels, and FSTL1 supplementation reduced myocardial MDA and 4-HNE levels in DOX-treated mice (Figures 4(g) and 4(h)).

### 3.5. FSTL1 Supplementation Reduced Cardiac Apoptosis in DOX-Treated Hearts

**Figure 5:**
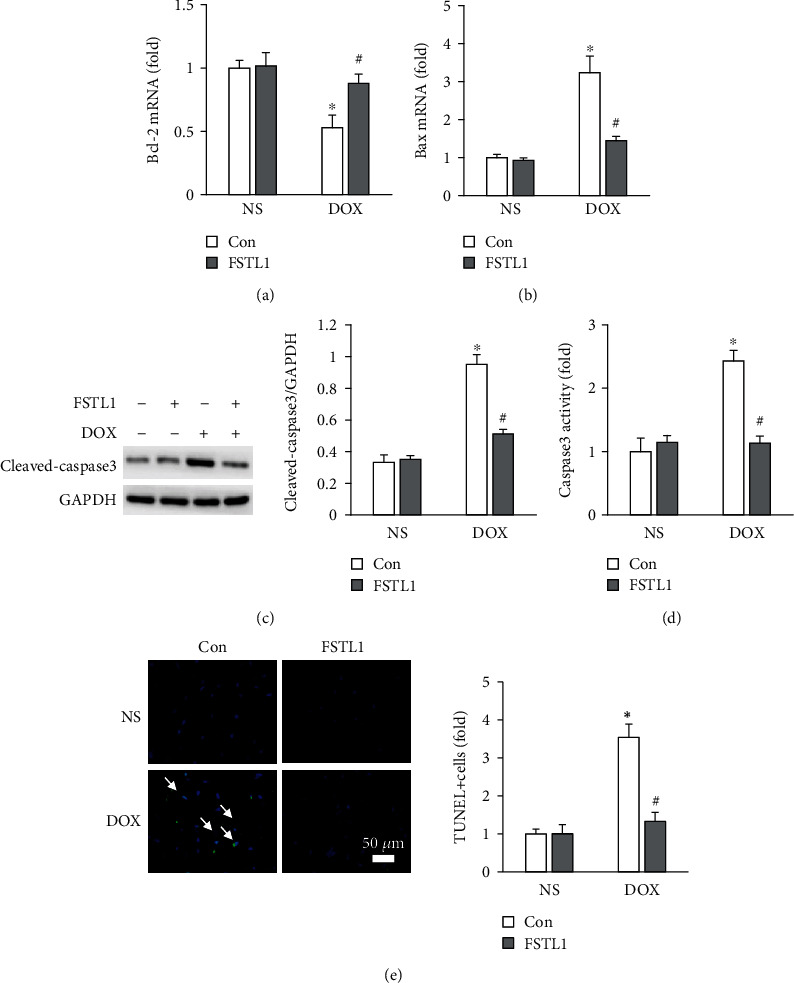
FSTL1 overexpression attenuated DOX-induced myocardial apoptosis. (a, b) The mRNA levels of Bcl-2 and Bax in the hearts (*n* = 6). (c, d) Cleaved caspase-3 and caspase-3 activity in the heart (*n* = 6). (e) TUNEL staining (*n* = 6). The data are expressed as the mean ± SD. ^∗^*P* < 0.05 vs. the NS/con group; ^#^*P* < 0.05 vs. the DOX/con group. The data were analyzed using one-way ANOVA, followed by Tukey's post hoc analysis.

Next, we detected whether FSTL1 could decrease DOX-related cardiac apoptosis in mice. FSTL1 upregulated the Bcl-2 mRNA level but decreased the Bax mRNA level in DOX-treated hearts (Figures 5(a) and 5(b)). DOX treatment upregulated the protein level of cleaved caspase-3; however, this pathological alteration was attenuated by FSTL1 supplementation (Figure 5(c)). Further evaluation also found that FSTL1 supplementation decreased caspase-3 activity in DOX-treated hearts (Figure 5(d)). To further confirm the antiapoptotic effect of FSTL1, we used the TUNEL assay to assess cardiac apoptosis. FSTL1 supplementation substantially decreased the number of TUNEL-positive cells in DOX-treated mice (Figure 5(e)).

### 3.6. Nrf2 Deficiency Antagonized FSTL1-Mediated Protection

**Figure 6:**
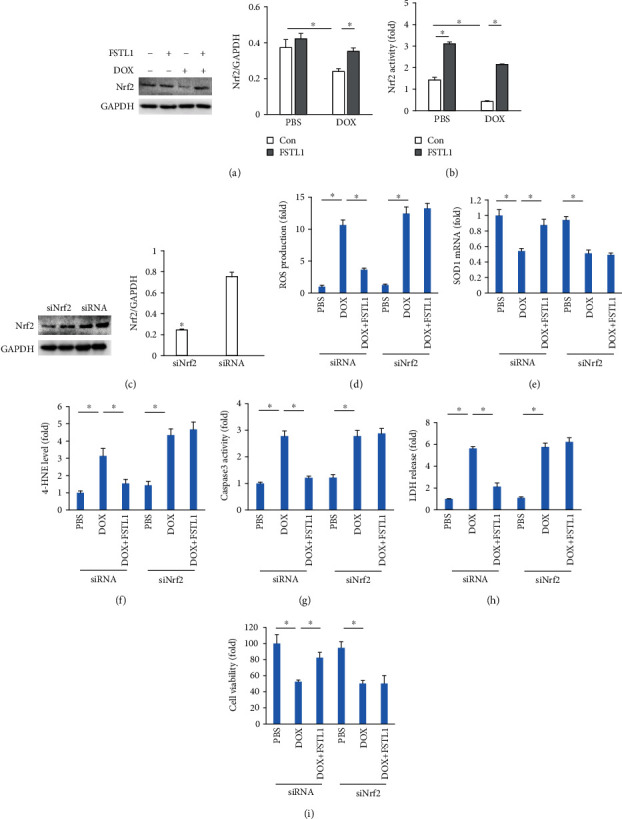
FSTL1 overexpression attenuated DOX-induced damage in vitro. (a, b) Nrf2 expression and Nrf2 activity in NRCMs (*n* = 6). (c) Nrf2 expression after siRNA infection (*n* = 6). (d) ROS production in NRCMs (*n* = 6). (e) The mRNA levels of SOD1 in NRCMs (*n* = 6). (f) The level of 4-HNE in NRCMs (*n* = 6). (g, h) Caspase 3 activity and LDH release (*n* = 6). (i) Cell viability (*n* = 6). ^∗^*P* < 0.05. For (c), the data were analyzed using two-tailed Student's *t*-tests. For the others, the data were analyzed using one-way ANOVA, followed by Tukey's post hoc analysis.

**Figure 7:**
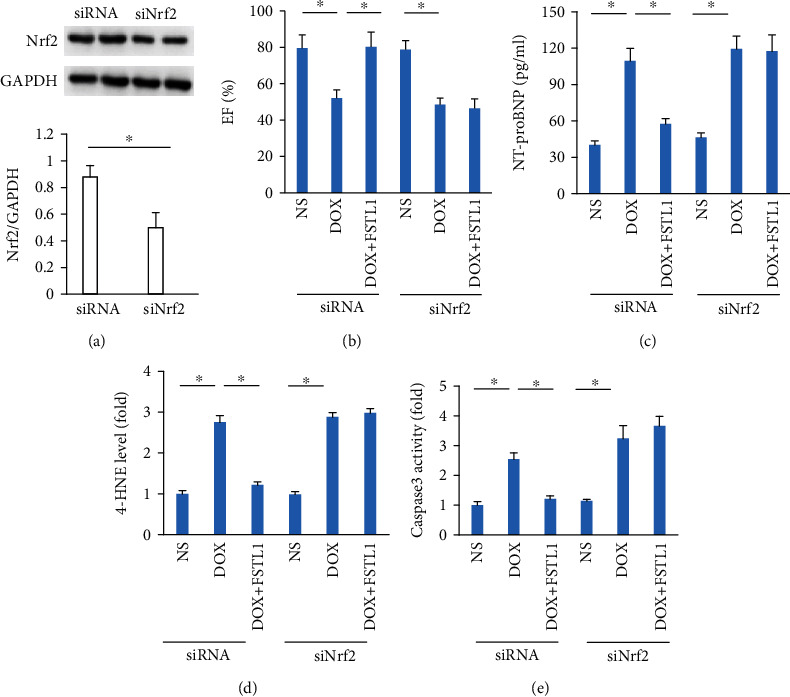
FSTL1 lost its cardioprotective effect in Nrf2-deficient hearts. (a) Nrf2 expression after siRNA infection (*n* = 6). (b) Ejection fraction (*n* = 6). (c) The level of NT-proBNP (*n* = 6). (d) The level of 4-HNE (*n* = 6). (e) Caspase-3 activity in the hearts (*n* = 6). ^∗^*P* < 0.05. For (a), the data were analyzed using two-tailed Student's *t*-tests. For the others, the data were analyzed using one-way ANOVA, followed by Tukey's post hoc analysis.

In view of the restoration of Nrf2 by FSTL1 in vivo, we measured Nrf2 protein expression in vitro. FSTL1 supplementation also significantly increased Nrf2 protein expression in DOX-treated NRCMs (Figure 6(a)). FSTL1 enhanced Nrf2 activity even in the absence of any stimuli. FSTL1 also increased nuclear Nrf2 activity in DOX-treated cells (Figure 6(b)). Next, we confirmed the hypothesis that FSTL1 exerted its protective effects by activating Nrf2. FSTL1 supplementation in vitro attenuated DOX-induced ROS production, upregulated SOD1 mRNA levels, and blocked the production of 4-HNE in NRCMs. However, this protection was abolished by Nrf2 deficiency (Figures 6(c)–6(f)). The inhibitory effects of FSTL1 on caspase-3 activity and LDH release were also lost in Nrf2-deficient NRCMs (Figures 6(g) and 6(h)). FSTL1 improved cell viability after DOX treatment, and siNrf2 antagonized this effect of FSTL1 (Figure 6(i)). To further verify the hypothesis that FSTL1 exerted its protective effects via activation of Nrf2 in mice, we used siRNAs to knock down Nrf2 expression in the heart. Our data suggested that FSTL1 could not exert cardioprotective effects in DOX-treated hearts, as indicated by the alterations in EF, plasma NT-proBNP, cardiac 4-HNE, and caspase-3 activity in mice (Figures 7(a)–7(e)).

## 4. Discussion

We showed for the first time that FSTL1 expression was decreased in DOX-treated hearts and that FSTL1 overexpression prevented acute DOX-induced injury in the heart. We found that FSTL1 overexpression prevented DOX-related cardiac atrophy, oxidative damage, and apoptosis, thus improving cardiac function. We also found that FSTL1 exerted cardioprotective effects by activating the Nrf2 signaling pathway in mice. Our study provide experimental evidence that FSTL1 possesses the potential to be a drug that can limit DOX-caused toxicity.

The pathogenesis of DOX-related cardiomyopathy is still unclear. Moreover, heart transplantation is the only approach for serious DOX-induced injury [2]. Therefore, devising a novel strategy to block DOX-induced cardiomyopathy would have tremendous benefits in clinical practice. Previous studies have found that several natural products that act as ROS scavengers have the ability to suppress acute cardiotoxicity caused by DOX, but there has been little success in clinical trials due to the low bioavailability of these drugs and secondary reactions with other molecules [15–17]. FSTL1 also inhibited pressure overload-induced cardiac hypertrophy in mice [18]. Another study found that FSTL1 alleviated myocardial damage caused by ischemia-reperfusion [5]. In the present study, we found that FSTL1, which was originally identified in a murine osteoblastic cell line, prevented DOX-induced body weight loss and cardiac atrophy and reduced cardiac injury marker release, thus improving cardiac function in mice. Moreover, overexpression of FSTL1 did not affect cardiac function or body weight in mice in the absence of DOX treatment. These observations suggest that FSTL1 might be a potential drug that can treat DOX-related cardiac injury.

One of the landmarks in acute cardiotoxicity caused by DOX is acute inflammation [14]. A previous study found that inhibition of myocardial inflammation accumulation suppressed DOX-related cardiac apoptosis in mice [4]. We also detected alterations in cardiac inflammation after FSTL1 overexpression, and our data showed that FSTL1 did not restrict myocardial inflammation in DOX-treated hearts, suggesting that the cardioprotective effect of FSTL1 was not dependent on attenuation of inflammation. It has been reported that free radical lesions are closely involved in DOX-related cardiac damage [19]. Overexpression of catalase or metallothionein could suppress DOX-related cardiotoxicity in mice [20, 21], suggesting a key role of oxidative damage in DOX-related cardiac injury. Highly reactive DOX metabolites induce mitochondrial injury and 4-HNE production, which could modify several mitochondrial proteins [22]. In the present study, we found that FSTL1 restored cardiac Nrf2 protein expression and SOD activity and reduced cardiac 4-HNE and MDA production in DOX-induced mice. Moreover, we found that FSTL1 restored cardiac mitochondrial complex activities to normal levels in mice treated with DOX. Moreover, we found that FSTL1 lost its cardioprotective effect against oxidative stress in vitro and in vivo after Nrf2 deficiency, suggesting that FSTL1 protects against DOX-related cardiac injury by activating the Nrf2 signaling pathway.

There is a direct correlation between the degree of myocardial apoptosis and the severity of DOX-induced cardiac injury [23]. Apoptotic cell death is a typical feature of DOX-induced cardiotoxicity. DOX induces cardiomyocyte apoptotic cell death by activating caspase-3 in vivo [24]. DOX treatment results in cytochrome C release and cardiomyocyte apoptosis and changes the Bcl-2/Bax ratio in mice [25]. Moreover, suppressing the degree of myocardial apoptosis could prevent DOX-related cardiac injury in mice [3]. In the present study, we found that FSTL1 overexpression upregulated Bcl-2 mRNA but decreased Bax mRNA levels, reduced cleaved-caspase3 protein expression, and decreased caspase3 activity in mice. Moreover, we found that FSTL1 lost these antiapoptotic effects in vitro and in vivo after Nrf2 deficiency, suggesting that FSTL1 protects against DOX-related apoptosis by activating the Nrf2 signaling pathway.

Overall, our results suggest that FSTL1 protects against DOX-induced acute toxicity by activating Nrf2 to suppress oxidative damage and apoptotic cell death, thus improving cardiac function. The present findings suggest that FSTL1 supplementation might represent a new cardioprotective strategy against DOX-induced cardiotoxicity.

## Data Availability

The data that support the findings of this study are available from the corresponding author upon reasonable request.
